# Prolonged cigarette smoke exposure alters mitochondrial structure and function in airway epithelial cells

**DOI:** 10.1186/1465-9921-14-97

**Published:** 2013-10-02

**Authors:** Roland F Hoffmann, Sina Zarrintan, Simone M Brandenburg, Arjan Kol, Harold G de Bruin, Shabnam Jafari, Freark Dijk, Dharamdajal Kalicharan, Marco Kelders, Harry R Gosker, Nick HT ten Hacken, Johannes J van der Want, Antoon JM van Oosterhout, Irene H Heijink

**Affiliations:** 1 Department of Pathology and Medical Biology, Laboratory of Allergology and Pulmonary Diseases, University of Groningen, University Medical Center Groningen, Groningen, The Netherlands; 2 Department of General Surgery, Tabriz University of Medical Sciences, Tabriz, Iran; 3 Department of Cell Biology, Groningen University, University Medical Center Groningen, Groningen, The Netherlands; 4 GRIAC Research Institute, University of Groningen, University Medical Center Groningen, Groningen, The Netherlands; 5 Department of Pulmonology, University of Groningen, University Medical Center Groningen, Groningen, The Netherlands; 6 Department of Respiratory Medicine, NUTRIM School for Nutrition, Toxicology and Metabolism, Maastricht University Medical Center+, Maastricht, The Netherlands; 7 Department of Laboratory Medicine, Electron Microscopy and Histology, Children’s and Women’s Health, Norwegian University of Science and Technology, Trondheim, Norway

**Keywords:** Mitochondria, Primary bronchial epithelial cells, Smoking, Reactive oxygen species, COPD

## Abstract

**Background:**

Cigarette smoking is the major risk factor for COPD, leading to chronic airway inflammation. We hypothesized that cigarette smoke induces structural and functional changes of airway epithelial mitochondria, with important implications for lung inflammation and COPD pathogenesis.

**Methods:**

We studied changes in mitochondrial morphology and in expression of markers for mitochondrial capacity, damage/biogenesis and fission/fusion in the human bronchial epithelial cell line BEAS-2B upon 6-months from ex-smoking COPD GOLD stage IV patients to age-matched smoking and never-smoking controls.

**Results:**

We observed that long-term CSE exposure induces robust changes in mitochondrial structure, including fragmentation, branching and quantity of cristae. The majority of these changes were persistent upon CSE depletion. Furthermore, long-term CSE exposure significantly increased the expression of specific fission/fusion markers (Fis1, Mfn1, Mfn2, Drp1 and Opa1), oxidative phosphorylation (OXPHOS) proteins (Complex II, III and V), and oxidative stress (Mn-SOD) markers. These changes were accompanied by increased levels of the pro-inflammatory mediators IL-6, IL-8, and IL-1β. Importantly, COPD primary bronchial epithelial cells (PBECs) displayed similar changes in mitochondrial morphology as observed in long-term CSE-exposure BEAS-2B cells. Moreover, expression of specific OXPHOS proteins was higher in PBECs from COPD patients than control smokers, as was the expression of mitochondrial stress marker PINK1.

**Conclusion:**

The observed mitochondrial changes in COPD epithelium are potentially the consequence of long-term exposure to cigarette smoke, leading to impaired mitochondrial function and may play a role in the pathogenesis of COPD.

## Introduction

Chronic Obstructive Pulmonary Disease (COPD), a chronic respiratory disease, is one of the leading causes of death today with a worldwide increase in incidence. COPD is characterized by accelerated lung function decline and a chronic inflammatory response in the lungs in response to cigarette smoke, the largest risk factor for COPD. Inhaled cigarette smoke first encounters the airway epithelium, where it may induce oxidative stress by both acute effects of its reactive components and by the intracellular generation of endogenous reactive oxygen species (ROS) by mitochondria [1-6]. Mitochondria can protect themselves and the cell from oxidative damage in several ways, e.g. by producing anti-oxidant scavengers, regulating the oxidative phosphorylation (OXPHOS) process responsible for ATP generation and exchanging mitochondrial DNA (mtDNA) through fusion and fission events [4,7-9]. Excessive oxidative stress and/or an imbalance or depletion of key mitochondrial fission and fusion markers, including Dynamin-related protein 1 (Drp1), Mitochondrial fission 1 protein (Fis1), Mitofussion (Mfn1 and Mfn2), Optic Atrophy 1 (OPA1) and mitochondrial transcription factor A (Tfam) can lead to mitochondrial damage and disorganized and aberrant cristae formation [10]. Furthermore, it will augment ROS production and cellular apoptosis through cytochrome-C release [8,11-16]. Normally, damaged mitochondria are repaired or cleared by a process called mitophagy, which is induced by PTEN-induced putative kinase 1 (PINK1). PINK1 regulates mitochondrial turnover and thereby protects mitochondria from stress, in concert with peroxisome proliferator-activated receptor gamma co-activator 1-alfa (PPARGC1α), which controls mitochondrial biogenesis [17-20]. We hypothesize that an aberrant response to cigarette smoke and excessive oxidative stress, e.g. due to an inefficient anti-oxidant response as observed in COPD lungs [21,22], may lead to impaired mitochondrial structure and function due to impaired clearance or repair. This may contribute the COPD pathology, inducing apoptosis, tissue damage as well as airway inflammation, since airway epithelial cells respond to increased ROS levels by producing cytokines/chemokines that attract inflammatory cells to the area of damage [5,6,21,23,24]. Although oxidative stress is known to induce post-translational modifications/damage to mitochondrial proteins [25,26], it is currently unknown whether mitochondrial structure and function in airway epithelial cells are affected by cigarette smoke [27,28], and whether these changes are present in COPD epithelium. Since COPD is a gradually acquired disease that develops upon chronic cigarette smoke exposure, we anticipated that a long-term cigarette smoke exposure model reflects the in vivo situation better than single exposure experiments. Therefore, we studied long-term (6 months) cigarette smoke extract (CSE)-induced changes in the human bronchial epithelial cell line BEAS-2B to mimic the condition of continuous smoking for prolonged periods, and investigated whether these changes are persistent upon CSE depletion. Additionally, we investigated whether similar changes are observed in mitochondria from primary bronchial epithelial cells (PBECs) of ex-smoking GOLD stage IV COPD patients with age-matched never-smoking and smoking controls.

## Methods

### Long-term CSE exposed human bronchial epithelial cell culture

BEAS-2B cells were grown in RPMI 1640 (Lonza, Walkersville, MD) supplemented with 15% heat-inactivated Fetal Bovine Serum (FBS), penicillin (100 U/ml) (Lonza) and streptomycin (100 μg/ml) (Lonza) on collagen-coated plates. Cells were grown to >90% confluence and passaged by trypsin. Cigarette smoke extract (CSE) was prepared fresh (used within 1 hr) from filter-less research-grade cigarettes (3R4F, Tobacco Research Institute, University of Kentucky, Lexington, KY) as described previously [5]. BEAS-2B cells were cultured in medium without 0% (control), 1%, 2.5%, 10% or 12.5% CSE for 6 months. For 10% CSE and 12.5% CSE the concentration was increased stepwise by 0.5-1% after each passage every 3–4 days starting from 2.5% CSE. Additionally, cells were exposed to 10% CSE for 3 months followed by 3 months culture in 0% CSE (referred to as -10% CSE). Cell viability was tested by trypan blue staining.

### Primary epithelial cell culture

Primary bronchial epithelial cells (PBECs) were isolated from ex-smoking GOLD stage IV COPD patients and age-matched never-smoking and smoking controls. Patient characteristics are described in Table 1[51]. The study was approved by the Medical Ethics Committee of the University Medical Center of Groningen and all subjects gave their written informed consent. PBECs were cultured in bronchial epithelium growth medium (Lonza) in flasks coated with collagen and fibronectin for at least 3 weeks in total as described [29]. Cells were plated in 24- or 6-well plates (EM), grown to confluence and placed overnight in BEBM containing transferrin, insulin, gentamicin and amphotericin B (Sigma-Aldrich).

**Table 1:** Characteristics of the subjects: primary bronchial epithelial cells (PBECs)

**Subject**	**Control non-smoker**	**Control smoker**	**GOLD stage IV**
**n**	15	15	24
**Age**	55,5 (43–76)	51.6 (40–70)	59,7 (48–71)
**Gender M (%)**	7 (46.7%)	12 (80%)	10 (41.7%)
**Pack-years**	0 (0–0)	31.3 (11–60)	39.3 (11–80)

All PBECs included in the study were provided by Lonza or the NORM and TIP study within the University Medical Center Groningen. COPD patients were included on a basis of FEV_1_ < 50% of predicted, FEV_1_/FVC < 70% and ≥20 pack-years for GOLD stage IV. All control subjects had FEV/FVC > 70% and FEV_1_ > 90% of predicted. FEV/FVC, FEV_1_ predicted and pack-years from Lonza cells are unknown.

### RT-qPCR

RNA isolation, Primer Sequences, RT-PCR on Tfam, OPA1, Drp1, Fis1, Mfn1, Mfn2 using MyiQ single-color Real-Time thermal cycler [30] and RT-PCR for IL-6, IL-8, IL-1β, Mn-SOD, PINK1, PPARGC1α, OPA1 and TFAM using Taqman® were performed as described in the online data Additional file 1.

### IL-8 release

Upon 3 days of culturing, levels of IL-8 were analyzed in cell-free culture supernatants by sandwich ELISA (R&D Systems, Abingdon, UK) according to the manufacturer’s instructions.

### Western blotting

Cell lysates were prepared and immunodetection was performed as described [29]. MitoProfile total OXPHOS antibody cocktail (Mitosciences, Eugene OR) or antibodies to mitochondrial loading control COXIV (clone 3E11) and anti-oxidant Mn-SOD (EMD Millipore Corporation, Billerica MA). Anti-GAPDH and Anti-β-actin (Cell Signalling Technology, Danvers MA, USA) were used for loading control.

### Electron microscopy

Cultured cells were fixed on 6-wells plates using 0.1 M phosphate buffered glutaraldehyde (2%) overnight at 4°C. Cells were pre-washed in 0.1 M cacodylate buffer and post-fixed in 1% OsO4 supplemented with 1.5% K3Fe(Cn6) for 2 hours, dehydrated and embedded in epoxy resin according to routine procedures. Sections were collected on copper grids, counterstained and inspected in a Philips CM 100. Data sampling is explained in online Additional file 1.

### Statistics

ANOVA and Chi-square was used to compare data sets indicated in Tables 2 and 3. Differences in protein/gene expression in BEAS-2B cells were tested by One-way ANOVA with Bonferroni post-hoc test or unpaired t-test. Differences between subject groups were analyzed by the Mann–Whitney test.

**Table 2:** Mitochondrial shape variation and density of the matrix

	**Shape**			**Status of matrix**	
	**Rod**	**Branching**	**Fragmented**	**Clear**	**Dense (Lipoid)**
**Control**	28 (93.3%)	0 (0.0%)	2 (6.7%)	30 (100.0%)	0 (0.0%)
**1% CSE**	25 (80.6%)	6 (19.4%)	0 (0.0%)	31 (100.0%)	0 (0.0%)
**2.5% CSE**	31 (96.9%)	0 (0.0%)	1 (3.1%)	28 (87.5%)	4 (12.5%)
**10% CSE**	24 (70.6%)	2 (5.9%)	8 (23.5%)	23 (67.6%)	11 (32.4%)
**12.5% CSE**	24 (69.6%)	3 (8.6%)	8 (22.9%)	29 (82.9%)	6 (17.1%)
**-10% CSE**	19 (63.3%)	9 (30.0%)	1 (3.1%)	22 (73.3%)	8 (26.7%)
	***			***	

***Estimated by chi-square test (p < 0.001).

BEAS-2B cells were exposed to various concentrations of cigarette smoke extract (CSE) for 6 months and subjected to EM analysis. BEAS-2B cells are exposed to: 0% CSE (control), 1% CSE, 2.5% CSE, 10% CSE, 12.5% CSE and -10% CSE (depletion). In total of 30–35 mitochondria were analyzed per experimental condition. Significance was determined by chi-square test.

**Table 3:** Frequencies and percentages of number of cristae and number of cristae divided by length

	**Number of cristae per mitochondrion per section**					FRQ/L**
	**0**	**< 3**	**3-6**	**>6**	**Mean ± SD**	**Mean ± SD**
**Control**	0 (0.0%)	0 (0.0%)	2 (6.7%)	28 (93.3%)	2.9 ± 0.3	2.2 ± 0.8
**1% CSE**	0 (0.0%)	2 (6.5%)	9 (29.0%)	20 (64.5%)	2.6 ± 0.6	1.9 ± 0.6
**2.5% CSE**	4 (12.5%)	4 (12.5%)	8 (25%)	16 (50%)	2.1 ± 1.1*	1.7 ± 1.0*
**10% CSE**	4 (11.8%)	5 (14.7%)	19 (55.9%)	6 (17.6%)	1.8 ± 0.9*	1.3 ± 0.8*
**12.5% CSE**	0 (0.0%)	5 (14.3%)	18 (51.4%)	12 (34.3%)	2.2 ± 0.7*	1.6 ± 0.6*
**-10% CSE**	1 (3.3%)	4 (13.3%)	12 (40%)	13 (43.3%)	2.2 ± 0.8*	1.6 ± 0.7*
	***				****	****

*p < 0.05 pairwise comparison to 0% CSE (Control).

**Frequency divided by length.

***Estimated by chi-square test (p < 0.001).

****Estimated by One-Way ANOVA test (p < 0.001).

**Figure 1:**
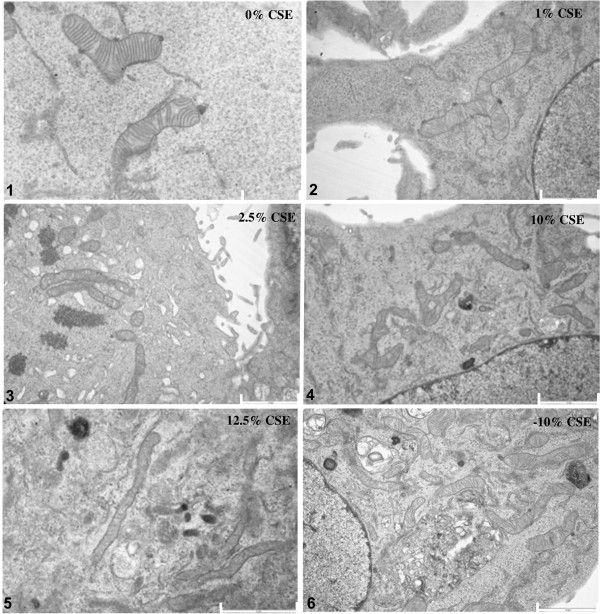
**Differences in mitochondrial shape, the status of matrix, swelling, visibility of double membranes, fragmentation, branching and cristae depletion upon long term CSE exposure in BEAS-2B cells.** BEAS-2B cells were exposed to various concentrations of cigarette smoke extract (CSE) for 6 months and subjected to EM analysis. Representative images of BEAS-2B cells exposed to: 0% CSE (control), 1% CSE, 2.5% CSE, 10% CSE, 12.5% CSE and -10% CSE. Scale bar indicates 2 μm and 1 μm (Top left).

BEAS-2B cells were exposed to various concentrations of cigarette smoke extract (CSE) for 6 months and subjected to EM analysis. Representative images of BEAS-2B cells exposed to: 0% CSE (control), 1% CSE, 2.5% CSE, 10% CSE, 12.5% CSE and -10% CSE are shown in Figure 1. In total of 30–35 mitochondria were analyzed per experimental condition. Significance was determined by chi-square test and One-Way ANOVA.

## Results

### Morphological ultrastructure and matrix changes in mitochondria of long-term CSE exposed

#### BEAS-2B cells

BEAS-2B cells exposed to different concentrations of CSE did not display differences in viability or morphology compared to cells that were not exposed to CSE, and were able to form a confluent layer (Online Additional file 1). Moreover, expression of the cellular senescence marker p21 was not affected (Online Additional file 1). Analysis of mitochondrial ultrastructure in long-term CSE exposed BEAS-2B cells revealed significant changes in morphological structure (branching/fragmentation), cristae formation and matrix density of mitochondria compared to the 0% CSE control cells (Figure 1). At 1% CSE, we observed an increased number of branched mitochondria when compared to the absence of CSE as well as to higher CSE concentrations (10% and 12.5% CSE, Figure 1 and Table 2, p < 0.001). Fragmentation, however, was more prominent at higher concentrations (10% and 12.5% CSE). Interestingly, although mitochondrial fragmentation was reduced upon depletion of CSE (-10% CSE) when compared to 10% CSE, CSE-depleted cells still showed more branched mitochondria compared to control conditions. This indicates that CSE-induced fragmentation, but not branching, is reversible upon its depletion. In addition to these morphological changes, the mitochondrial matrix was significantly affected by 2.5%, 10% and 12.5% CSE (Table 2, p < 0.001), which remained present upon CSE depletion (-10%). The density of the “dark” lipoid matrix also increased with higher CSE concentrations and this increased density also remained present upon depletion of CSE (-10% CSE).

Subsequently, we analyzed the relative frequency of mitochondrial cristae that were observed within a single mitochondrion. We observed that the numbers of cristae were strongly reduced upon long-term CSE exposure, which was already observed at 1% CSE further diminished with increasing concentrations of CSE and remained present after CSE depletion (-10% CSE) (Table 2, p < 0.001) compared to the 0% CSE treated cells. Together, these data show that long-term CSE exposure induces strong and persistent structural mitochondrial changes. These data are further confirmed by fluorescent 3D imaging of mitochondrial distribution in BEAS-2B cells upon long-term 10% CSE exposure, showing increased numbers of branched mitochondria (Online Additional file 1).

### Transcriptional increase in OPA1 expression upon long-term CSE exposure in BEAS-2B cells

**Figure 2:**
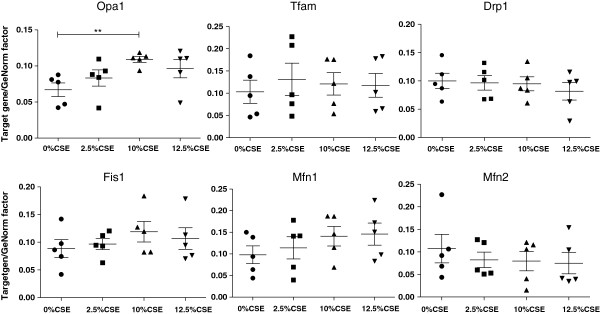
**Expression of fission and fusion markers in long-term CSE exposed BEAS-2B cells.** Cells were exposed to 0% CSE, 2.5% CSE, 10% CSE, 12.5% CSE (n = 5 for each group) for 6 months. RNA was isolated and fission and fusion markers were detected by qPCR. Ct values were obtained for the standard curve and each sample. Relative DNA starting quantities of samples were derived from the standard curve based on the Ct values using the iQ5 optical system software version 2.1. Genes of interest expression was normalized to a GeNorm factor obtained from the housekeeping genes RPLPO and RPL13a. Median interquartile ranges (IQR) are indicated. * = p < 0.05 between the indicated values.

Next, we performed qRT-PCR to examine whether the observed changes may result from altered mRNA expression of various genes involved in fission and fusion, e.g. Mfn1, Mfn2, Fis1, Drp1, OPA1 and the mitochondrial transcription factor Tfam (Figure 2). We observed a significant 1.9 (±0.1) fold increase in the mRNA expression of the profusion related protein OPA1 upon exposure to 10% CSE (Figure 2). Surprisingly, we observed no significant transcriptional differences in other mitochondrial fission (Fis1 and Drp1) or fusion markers (Mfn1 and Mfn2) nor in Tfam expression (Figure 2).

### Increased mitochondrial capacity in long-term CSE exposed BEAS-2B cells

**Figure 3:**
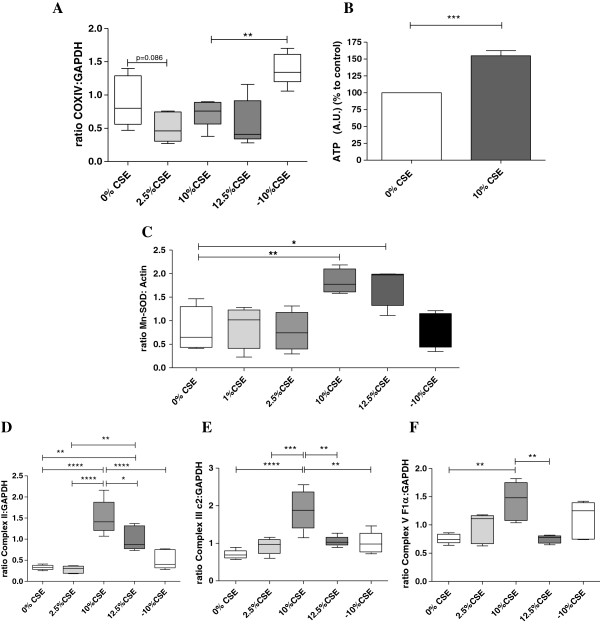
**Mitochondrial capacity is increased in long-term cigarette CSE exposed BEAS-2B cells.** Cells were exposed to 0% CSE, 2.5% CSE, 10% CSE 12.5% CSE (n = 4-5) for 6 months. **A)** Mitochondrial mass was determined by COXIV as a mitochondrial loading control using western blotting. GAPDH was used as loading control. **B)** ATP levels were measured by bioluminescence. **C)** Mn-SOD protein levels were detected by western blotting, related to actin and medians are indicated. Western blot analysis of, **D)** Complex II, **E)** Complex III and **F)** Complex V of the oxidative phosphorylation, related to GAPDH (medians are indicated). p = 0.06, p = 0.08, * = p < 0.05, ** = p < 0.01, *** = p < 0.001 and **** = p < 0.0001 between the indicated values.

To assess whether CSE exposure induces differences in the total mitochondrial fraction, we studied the expression of the mitochondrial COXIV protein (which is generally continuously expressed at a high level). Although exposure to 2.5%, 10% and 12.5% slightly decreased the COXIV levels, this was not significant when compared to the 0% CSE control cells (Figure 3A). However, upon CSE depletion, mitochondrial mass showed a marked and significant increase compared to the presence of 10% CSE, indicating a recovery process upon CSE depletion (Figure 3A). Next, we studied whether the observed structural changes may affect mitochondrial function by studying ATP levels after 10% CSE exposure as a proof of concept. Contradictory to the unaltered mitochondrial mass and reduced cristae number, however, ATP levels were increased in 10% CSE exposed cells by approximately 1.6 fold when compared to the control (0% CSE) condition (Figure 3B), which may reflect an increase in mitochondrial OXPHOS capacity. In line with this, exposure to 10% CSE and 12.5% CSE induced a ~2-3 fold increase protein levels of the anti-oxidant protein Manganese Superoxide Dismutase (Mn-SOD, Figure 3C), a marker of oxidative stress, with a similar increase in Mn-SOD expression upon exposure to 10% CSE and 12.5% CSE at the mRNA level (data not shown). Interestingly, Mn-SOD protein levels were restored in -10% CSE exposed cells indicating a reduction in oxidative stress levels upon CSE depletion. To study whether oxidative stress causes mtDNA damage, we assessed mtDNA damage by fragment PCR [31] and observed that hydrogen peroxide, gas phase cigarette smoke and CSE induced mtDNA damage in BEAS-2B cells [1,26,32,33] (Online Additional file 1).

Regulation of oxidative phosphorylation is important for mitochondrial capacity and the protection against oxidative stress. Therefore, we analyzed the expression of three major components of the OXPHOS system i.e., Complex II, Complex III core 2 and (the ATPase subunit) Complex V F1α by western blotting. Interestingly, Complex II (Figure 3D), Complex III (Figure 3E) and Complex V (Figure 3F) were significantly increased in cells cultured in 10% CSE, but not 2.5%, when compared to the other concentrations. Surprisingly, expression of all complexes was again lower upon 12.5% CSE when compared to 10% CSE exposure, although the expression of Complex II, but none of the other complexes, was still significantly increased at 12.5% CSE. CSE depletion resulted in the restoration of complex II and III protein levels, similar to the non-persistent effects of CSE on mitochondrial mass and Mn-SOD, while complex V levels were not significantly reduced upon CSE depletion (Figure 3F).

### Increased inflammatory response in long-term CSE exposed cells

**Figure 4:**
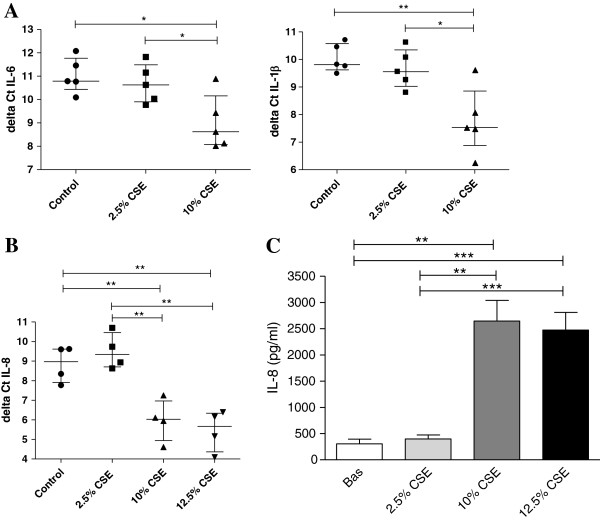
**Increased inflammatory response in long-term CSE exposed BEAS-2B cells.** Cells were exposed to 0% CSE, 2.5% CSE, 10% CSE 12.5% CSE as indicated for 6 months. **A)** IL-6, IL-1β mRNA expression (n = 5) and **B)** IL-8 mRNA expression was detected by qPCR and related to the housekeeping genes (n = 4). ΔCt values are shown (lower values reflect higher expression) and median interquartile ranges (IQR) are indicated. **C)** IL-8 protein levels were measured in cell-free culture supernatants (n = 4) and mean (±SEM) levels are shown. Significance is as indicated, * = p < 0.05, ** = p < 0.01, *** = p < 0.001.

To investigate whether these mitochondrial changes induced by long-term CSE exposure were accompanied by increased pro-inflammatory activity of bronchial epithelium, we measured expression of the pro-inflammatory cytokines IL-1β, IL-6 and IL-8, which are known to be elevated in the airways of COPD patients [34,35]. We found a significant increase in IL-1β and IL-6 mRNA expression in cells exposed to 10% CSE compared to control cells (Figure 4A). Additionally, IL-8 mRNA (Figure 4B) and protein levels (Figure 4C) were significantly increased in cells exposed to 10% and 12.5% CSE. CSE in a concentration of 2.5% did not to affect the mRNA nor on protein expression of pro-inflammatory cytokines, in line with the absence of effects on mitochondrial function (Figure 4A, 4C).

### Mitochondrial changes in PBECs from COPD patients compared to PBECs from (never-) smoking individuals

**Figure 5:**
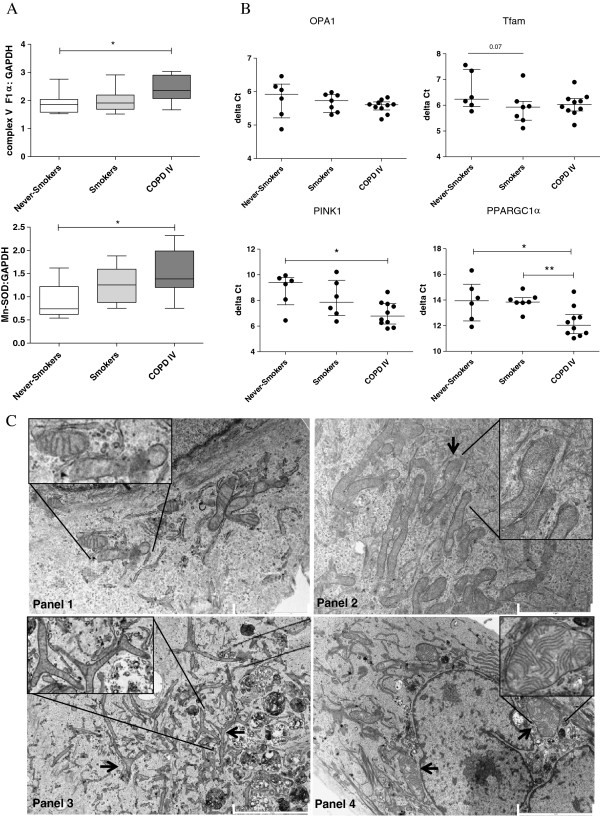
**Primary bronchial epithelial cells (PBECs) from COPD patients display elongated and swollen mitochondria, fragmentation, branching and cristae depletion and altered expression of mitochondrial markers compared to control PBECs.** PBECs were isolated from ex-smoking COPD patients with GOLD stage IV (n = 10), control smokers (n = 7) and never-smokers (n = 6) **A)** Complex V ATPase protein and Mn-SOD were detected by western blotting. GAPDH was used as loading control. **B)** OPA1, Tfam, PINK1 and PPARGC1α mRNA expression was detected by qPCR and related to housekeeping genes. ΔCt values are shown and median interquartile ranges (IQR) are indicated. **C)** Panel 1 shows a representative picture of PBECs from 4 never-smokers displaying normal mitochondria. Panel 2 shows a representative picture from 9 ex-smoking COPD patients displaying increased numbers of mitochondria with cristae depletion and ’club shaped’ ends. Panel 3 COPD patient shows severe branching. Panel 4 COPD patient shows swollen structures. Scale bar indicates 5 μm (bottom) and 2 μm (Top). p = 0.07, * = p < 0.05 and ** = p < 0.01 between the indicated values.

In order to assess whether long-term smoking also results in (persistent) structural changes in epithelial mitochondria in COPD, we studied mitochondrial alterations in PBECs derived from severe ex-smoking COPD GOLD stage IV patients and age-matched never-smoking and smoking controls. First, we investigated OXPHOS levels and observed that protein levels of Complex V F1α were not significantly different in PBECs from non-smokers compared to smokers. Interestingly, levels were significantly increased in PBECs from COPD patients when compared to non-smoking control PBECs, while a trend was visible when compared to PBECs from smokers, indicating a disease-related effect (Figure 5A). Similar to the data in BEAS-2B, increased Complex V levels were accompanied by increased Mn-SOD protein levels in PBECs from COPD patients, which may reflect a compensatory mechanism in response to increased energy demand and/or elevated oxidative stress (Figure 5A).

In contrast to the elevated levels in CSE-exposed BEAS-2B cells, we found no evidence for altered mRNA expression of OPA1 or Tfam in PBECs from COPD patients or control smokers compared to never-smokers (Figure 5B). We also studied the expression of PINK1 and PPARGC1α, genes that are activated upon mitochondrial damage and play a key role in mitochondrial turnover. Of interest, the mRNA expression of both PINK1, a marker not significantly different in CSE exposed BEAS-2B cells (Online Additional file 1), and PPARGC1α, a marker that was not detectable in CSE exposed BEAS-2B cells, was significantly increased in PBECs from COPD patients when compared to non-smoking controls (Figure 5B). In line herewith, we observed that PBECs from COPD patients show remarkable abnormalities in mitochondrial structure, similar to the changes observed in BEAS-2B cells upon long-term CSE-exposure, albeit more severe. These observations include excessive branching of the mitochondria and cristae depletion, but also mitochondrial swelling and elongation, when compared to mitochondria in control PBECs (Figure 5C). Together, our data indicate that epithelium from severe COPD patient’s displays persistent mitochondrial damage.

## Discussion

For the first time, we show that long-term CSE exposure induces robust and persistent changes in mitochondrial structure and function in human bronchial epithelial cells, including increased fragmentation, branching, density of the matrix and reduced numbers of cristae. All of these changes, except for fragmentation, remained present after depletion of CSE, suggesting that these are persistent upon smoking cessation. In line with the observed changes, mRNA expression of OPA1, a critical regulator of cristae formation and fission/fusion, was increased at the transcriptional level in long-term CSE-exposed BEAS-2B cells. Furthermore, we observed increased protein levels of OXPHOS components and increased levels of the oxidative stress marker and anti-oxidant Mn-SOD, indicating augmented energy demand and oxidative stress. These mitochondrial changes were accompanied by increased pro-inflammatory activity in long-term CSE-exposed cells, as reflected by higher levels of the pro-inflammatory cytokines IL-1β, IL-6 and IL-8. Moreover, our data show that similar mitochondrial changes are present in bronchial epithelium from severe ex-smoking COPD patients in comparison to healthy controls, with depletion of cristae, increased branching, elongation and swelling of the mitochondria, increased Mn-SOD, PINK1 and PPARGC1α, but not OPA1 mRNA expression and increased OXPHOS Complex VF1α levels. These changes in Mn-SOD, PINK1, PPARGC1α and Complex VF1α were not observed in epithelial cells from control smokers.

It is tempting to speculate that an attenuated anti-oxidant response with elevated ROS in COPD [21,22,28,36] may lead to an increased oxidant burden, possibly contributing to the observed mitochondrial defects in bronchial epithelial cells from COPD patients [6,32,37]. Cigarette smoking induces both oxidative stress and anti-oxidant responses in airway epithelium and, with great variability amongst individuals [38]. An increased oxidant burden due to lower levels of anti-oxidant defense has been reported in COPD, which has been associated with decreased lung function [39,40]. Of importance, oxidative stress exceeding the anti-oxidant response may also lead to accelerated ageing and thus accelerated lung function decline [10,22,27]. Recent studies suggest that due to increased oxidative stress, damaged mtDNA induces premature ageing in human cells [9,27,41]. Importantly, similar morphological changes of mitochondria as observed here are regarded as biomarkers of mitochondrial aging. For example, senescent endothelial cells showed degenerated cristae and swollen regions devoid of cristae [10]. Of note, the ageing lung shows morphological changes that resemble emphysema in COPD, including alveolar enlargement and a reduction in elastic recoil, contributing to lung function decline [42]. Although we have not studied effects in alveolar epithelial cells, our findings may also have important implications for lung emphysema. ROS are potent inducers of cellular apoptosis by promoting the release of cytochrome-C, which may lead to alveolar cell apoptosis, lung tissue damage, inflammation and emphysema [27,43-45]. Thus, we anticipate that lung epithelium from COPD patients may be more prone to cigarette smoking-induced mitochondrial damage/dysfunction and premature ageing, which may eventually affect lung function. Future studies will be of interest to assess whether the airway epithelial structure and function of mitochondria differs between in young non-smoking individuals who are non-susceptible and susceptible to develop COPD.

In line with the hypothesis that epithelium from control smokers is better protected against cigarette smoke-induced changes in mitochondrial function, OXPHOS complex levels were not (persistently) higher in PBECs from smokers with normal lung function than from never-smokers, although OXPHOS complex II, III and VF1α were increased upon long-term CSE exposure in BEAS-2B. With respect to PBECs from ex-smoking COPD patients, we only observed an increase in complex VF1α, but not complex II and III, in line with the finding that the levels of complex II and complex III returned back to baseline levels upon CSE depletion in BEAS-2B cells.

Our data strongly suggest that cigarette smoking induces structural and functional changes in mitochondria resulting in a pro-inflammatory phenotype of airway epithelium in severe COPD patients, and that these are persistent, as they are still present upon smoking cessation. In line with this, all CSE-induced morphological changes in BEAS-2B mitochondria that remained present upon CSE depletion were also observed in PBECs from COPD patients. Furthermore, PBECs from COPD patients displayed increased levels of Mn-SOD and OXPHOS complex VF1α, in agreement with the findings in BEAS-2B cells. These changes indicate increased cellular energy demand that is still present in cultured airway epithelium from ex-smoking COPD patients. Persistent mitochondrial damage in airway epithelium of COPD patients is further evidenced by the increased expression of PINK1, a sensor for damaged mitochondria, and PPARGC1α, a regulator of mitochondrial biogenesis [20,46]. Both PINK1 and the fission/fusion protein OPA1 are known to be involved in the regulation of cristae formation [47], and increased expression of these proteins may thus reflect cristae damage. Since we did not observe significant changes in PINK1 and PPARCG1α expression in control smokers, nor in the CSE-exposed BEAS-2B cells, we anticipate that additional factors, e.g. susceptibility gene expression, may be involved in their aberrant expression in COPD epithelium. Vice versa, we did not observe significant differences in OPA1 expression between the subject groups, although OPA1 expression was increased in the long-term CSE-exposed BEAS-2B cells. We did not observe significant differences in other important mitochondrial genome and fusion markers, including Tfam and Drp1, between the subject groups or upon CSE exposure in BEAS-2B. However, we cannot exclude the possibility that these markers are modified at the posttranslational level by cigarette smoke-induced oxidative stress, leading to the observed changes in mitochondrial morphology, and this needs further investigation.

**Figure 6:**
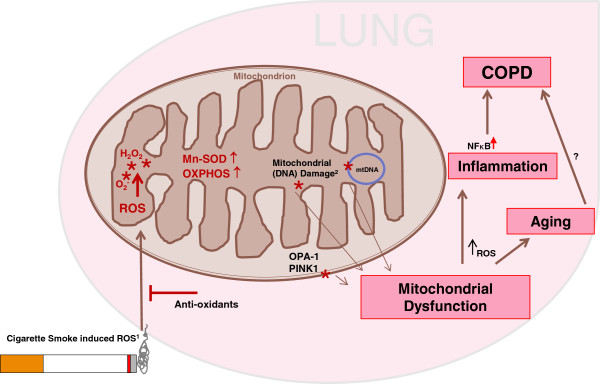
**Proposed model on the effect of cigarette smoke induced mitochondrial changes.** Cigarette smoke is able to generate intracellular ROS production of which mitochondria are the main producers. ROS can damage important cellular components like nuclear DNA and organelles. Additionally, endogenous ROS affect mitochondrial DNA, membrane lipids and proteins, including fission/fusion proteins and PINK1, and induce oxidative modification or blocking of the OXPHOS system. Upon persistent mitochondrial damage or oxidative stress exceeding the anti-oxidant response, mitochondrial dysfunction will be introduced. Mitochondrial dysfunction and ROS from cigarette smoke may eventually induce inflammation, premature ageing and the onset of COPD.

To summarize, we propose that mitochondrial structure changes in COPD epithelium may derive from a sustained oxidative stress due to cigarette smoke exposure, which results in an inflammatory response (Figure 6). In line with our findings on the increased expression of pro-inflammatory cytokines upon long-term epithelial CSE exposure, bronchial epithelial cell from COPD patients have been shown to produce higher levels of IL-8 than controls individuals [34,35]. Excessive oxidative stress may induce oxidative modifications, including peroxidation of mitochondrial lipids, oxidative damage of mitochondrial proteins including OXPHOS components, cristae remodeling, mutations and severe damage in mitochondrial DNA (mtDNA) [5,6,22,39,40,42,43]. This may result in a further increase in oxidative stress, with important consequences, since ROS are additionally known to increase the NF-κB response. Eventually, this pro-inflammatory activity of the airway epithelium [2,23,48-51] may contribute to the development of chronic airway inflammation in COPD.

In conclusion, our study demonstrates that long-term exposure to CSE leads to structural and functional changes in mitochondria that persist upon smoking cessation and may contribute to the onset pathogenesis of COPD. Herewith, we propose a new pathophysiological concept in the development of the COPD, which opens novel avenues for therapeutic strategies aimed towards the improvement of mitochondrial function and protection against ROS/damage.

## Abbreviations

CSE: Cigarette smoke extract; Drp1: Dynamin-related protein 1; Fis1: Mitochondrial fission 1 protein; Mfn1 and Mfn2: Mitofussion1 and 2; Mn-SOD: Manganese Superoxide Dismutase; Opa1: Optic atrophy type 1; OXPHOS: Oxidative phosphorylation; PBEC: Primary Bronchial Epithelial Cells; PINK1: PTEN-induced putative kinase 1; PPARGC1α: Proliferator-activated receptor gamma co-activator 1-alfa; ROS: Reactive Oxygen Species; Tfam: Mitochondrial transcription factor A.

## Competing interests

The authors declare that they have no competing interests.

## Authors’ contributions

RH designed and performed the experiments, performed statistical analysis and drafted the manuscript. AK performed experiments. SB performed experiments and helped with primary cell culture. HdB and MK performed the qPCRs. JvdW supported and supervised the Electron Microscopy analysis. SS, FD, DK, SJ participated in Electron Microscopy. AvO and IH participated in the design and coordination of the study. NtH participated in inclusion of the patients. IH helped to draft the manuscript. HG, JvdW, AvO and IH revised the manuscript. All authors read and approved the final manuscript.

## Supplementary Material

Additional file 1 Online supplementary data. Click here for file
