# Supplementary material for: Prolonged cigarette smoke exposure alters mitochondrial structure and function in airway epithelial cells
Source: Respir Res. 2013 Oct 2;14(1):97. doi: 10.1186/1465-9921-14-97 (PMC3852998; doi:10.1186/1465-9921-14-97)
Supplement: Additional file 1 — Online supplementary data. [file 1465-9921-14-97-S1.doc]

**Online supplementary data**

**Prolonged cigarette smoke exposure alters mitochondrial structure and function in airway epithelial cells**

*Roland F. Hoffmann^1,4^, Sina Zarrintan ^2,3^, Simone M. Brandenburg^1^, Arjan Kol^1^, Harold G. de Bruin^1,4^, Shabnam* *Jafari^3^, Freark Dijk^3^, Dharamdajal Kalicharan^3^, Marco Kelders^6^, Harry R. Gosker^6^, Nick H.T. ten Hacken^4,5^, Johannes J. van der Want^3,6^, Antoon J.M. van Oosterhout^1,4^, Irene H. Heijink^1,4,5^*

| Target | Bronseq | Fw primer | Rev primer |
| --- | --- | --- | --- |
| RPLPO | NM_001002 | TCTACAACCCTGAAGTGCTTGATATC | GCAGACAGACACTGGCAACATT |
| RPL13A | NM_012423 | CCTGGAGGAGAAGAGGAAAGAGA | TTGAGGACCTCTGTGTATTTGTCAA |
| B2M | ENSG00000166710 | CTGTGCTCGCGCTACTCTCTCTT | TGAGTAAACCTGAATCTTTGGAGTACGC |
| GAPDH | ENST00000229239 | GCACCACCAACTGCTTAGCA | TGGCAGTGATGGCATGGA |
| Cyclo | NM_021130 | CATCTGCACTGCCAAGACTGA | TTCATGCCTTCTTTCACTTTGC |
| Actin | NM_001101 | AAGCCACCCCACTTCTCTCTAA | AATGCTATCACCTCCCCTGTGT |
| Mfn1 | ENSG00000171109 | CTGAGGATGATTGTTAGCTCCACG | CAGGCGAGCAAAAGTGGTAGC |
| Mfn2 | ENSG00000116688 | TGGACCACCAAGGCCAAGGA | TCTCGCTGGCATGCTCCAC |
| Opa1 | ENSG00000198836 | TACCAAAGGCATTTTGTAGATTCTGAGTT | GCATGCGCTGTATACGCCAA |
| Fis1 | ENSG00000214253 | CCTGGTGCGGAGCAAGTACAA | TCCTTGCTCCCTTTGGGCAG |
| Drp1 | ENSG00000087470 | CGACTCATTAAATCATATTTTCTCATTGTCAG | TGCATTACTGCCTTTGGCACACT |
| MTP18 | ENSG00000242114 | TCTCTACCGGGACACGTGGGT | GAAAGCCTCGCCCACCTCA |
| PGC1a | ENSMUSG00000029167 | ATGCCTTTAGATGTGAGCTAACAGTAGGTAATGA | CGTACAGCCATCAAAAAGGGACAC |
| Tfam | ENSG00000108064 | GAAAGATTCCAAGAAGCTAAGGGTGATT | TCCAGTTTTCCTTTACAGTCTTCAGCTTTT |

**S1. Primer sequences and RT-qPCR methods**. RNA was extracted using TriReagent, Applied Biosystems/Ambion (Foster City, CA), reverse transcribed to cDNA using iScript cDNA Synthesis Kit (Bio-Rad, Herculus, CA). Primers (Sigma Genosys, Haverhill, UK) were designed using Primer Express 2.0 (Applied Biosystems, Foster City, CA). Real-time PCRs on Tfam, OPA1, Drp1, Fis1, Mfn1, Mfn2 were performed in a MyiQ single-color Real-Time thermal cycler (Bio-Rad, Herculus, CA). Normalization was performed to a GeNorm factor obtained from reference genes RPLPO, RPL13A, GAPDH, Cyclophilin and β-actin (Freeware, Gent University, Belgium)**.** IL-6, IL-8, IL-1β, histon deacetylase (HDAC)2, Mn**-**SOD, PINK1, PPARGC1α, OPA1 and TFAM expression was analyzed by real-time PCR using Taqman® (Applied Biosystems, Foster City, CA). Validated probes and housekeeping genes, B2M and PP1A and TaqMan Master Mix were purchased from Applied Biosystems. Amplification efficiencies were similar between the target and housekeeping genes. Samples were measured in duplicates, which never diverged more than 0.5 Ct.


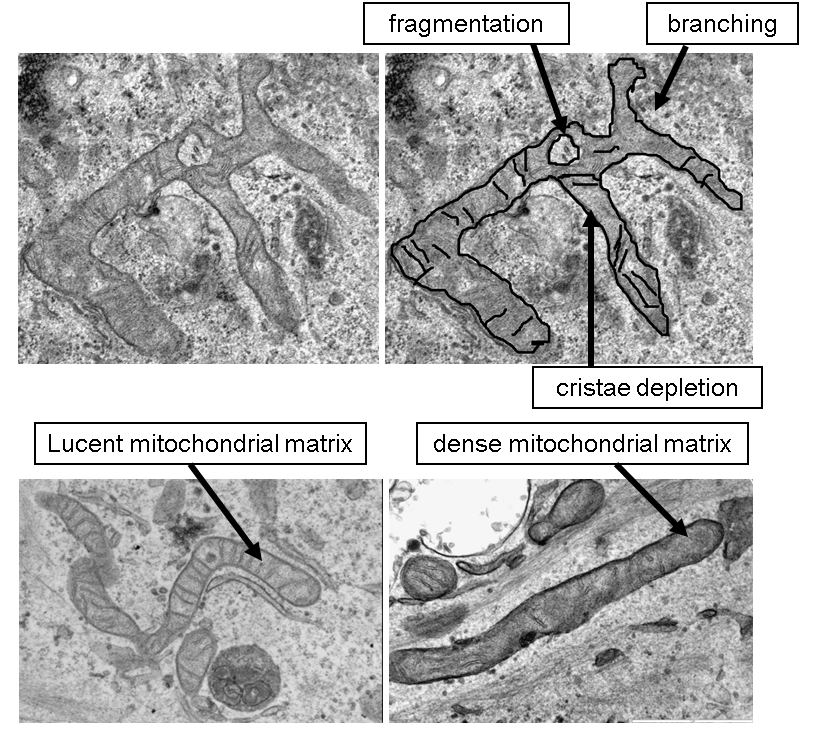


**S2. Image criteria; analysis of mitochondrial ultrastructure in in long-term CSE- exposed BEAS-2B cells.** We studied mitochondrial ultrastructural changes in long-term (6 months) CSE exposed BEAS-2B cells at different CSE concentrations (0%, 1%, 2.5%, 10% and 12.5%). Additionally, we exposed cells to 10% CSE for 3 months followed by 3 months control conditions (-10%) in order to determine whether or not the induced changes are permanent. For electron microscopy analysis, mitochondria were randomly selected from cells with intact cytoplasm and nuclei. Other parameters that were taken into account include distribution of mitochondria, either in clusters or dispersed. Images were taken of 3-5 mitochondria in a clear visible field at different magnification (4500x, 17500x) to a total of 30-35 mitochondria per experimental condition. Shape was measured with respect to length, or in case of branching with bifurcating extensions and a third category of fragmented mitochondria. The mitochondrial matrix was distinguished on the basis of electron lucent “transparent matrix” or an electron dense “dark” lipoid matrix. Since most mitochondria will not be viewed in their full extent due to their unknown orientation, we have calculated the frequency of the cristae per unit length; these values were also significantly different from a random distribution (Table 2, last column). Because mitochondrial cristae are equally distributed in a randomly oriented cell, we compared categories with respectively 0, <3, 3-6 and more than 6 cristae. Representative images of the mitochondrial changes are shown, including increased branching, fragmentation, density of the mitochondrial matrix and cristae depletion.

**BEAS-2B Control BEAS-2B 2.5% CSE BEAS-2B 10% CSE**


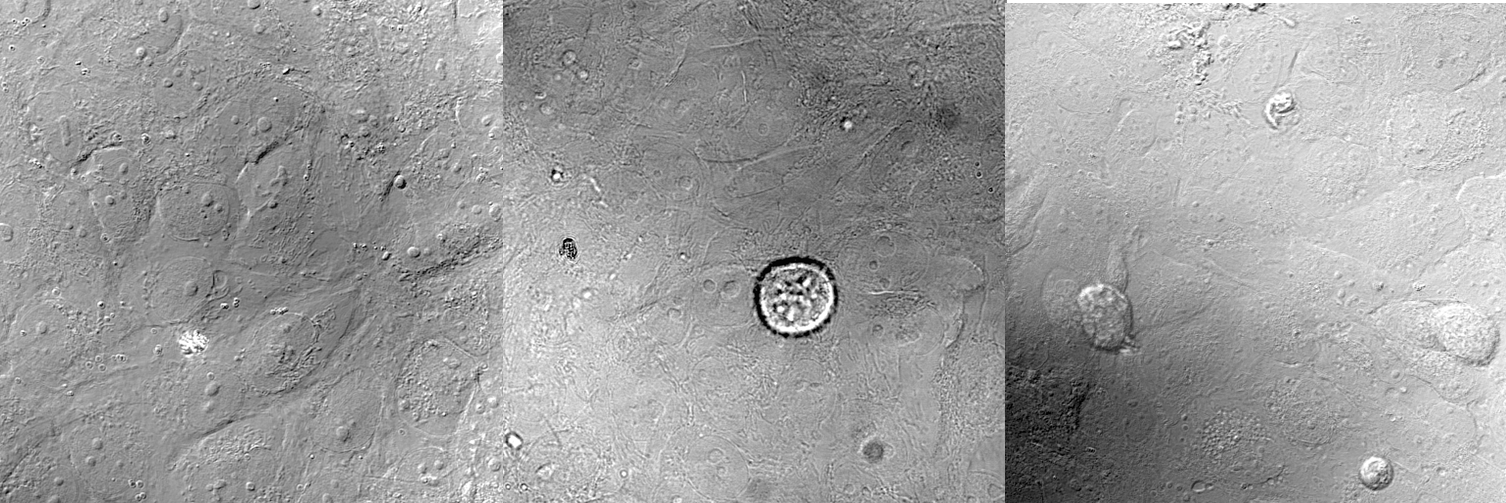
**A)**

**B)**

**S3. Long-term CSE-exposed BEAS-2B do not show abnormal morphology or senescence.** BEAS-2B cells grown for 6 months in 0%, 2.5% or 10% CSE. A) Bright-field microscopy images of BEAS-2B cells cultured for 6 months in the absence (0%=control) or presence of 2.5% or 10% CSE showed no abnormal cell growth during cigarette smoke exposure. B) mRNA expression of the senescence marker p21 does not significantly differ between control- and long-term CSE exposed BEAS-2B cells. ΔCt values are shown (lower values reflect higher expression) and median interquartile ranges (IQR) are indicated


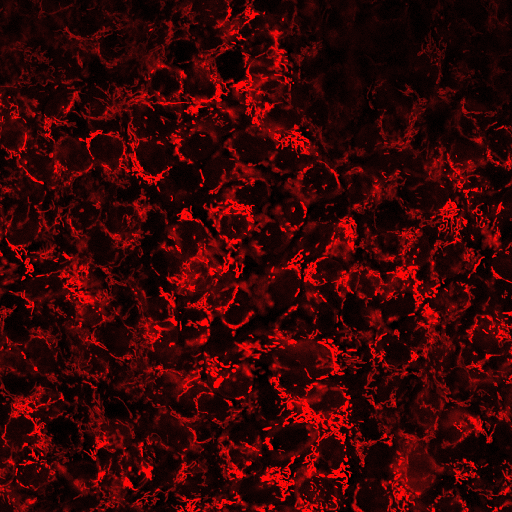

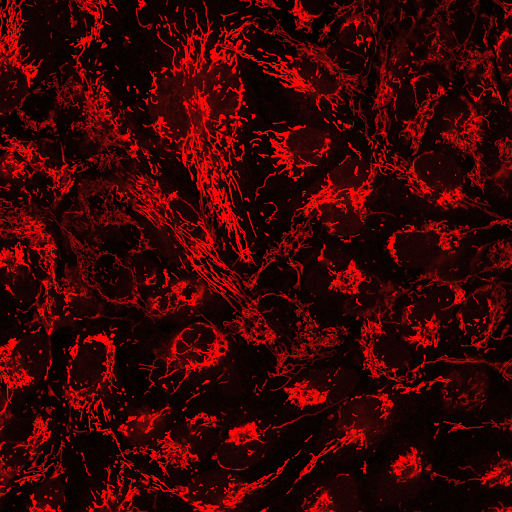

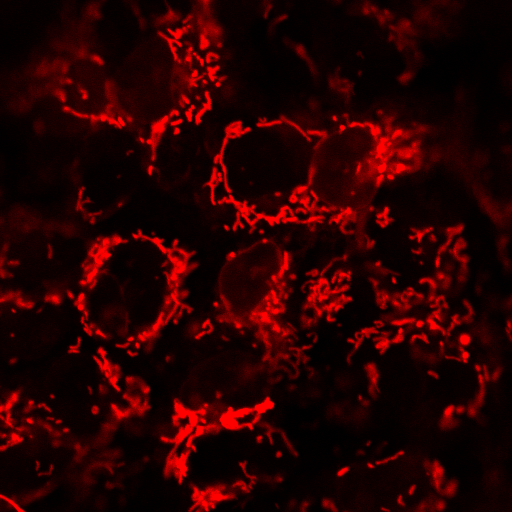

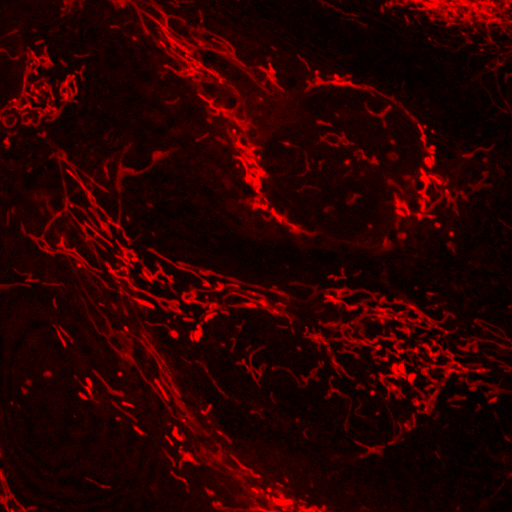


63 x 1.4 Leica AOBS

63 x 1.4 Leica AOBS

BEAS-2B 10% CSE

BEAS-2B 0% CSE

**S4.** **Increased numbers of branched mitochondria in long-term CSE exposed** **BEAS-2B cells.** BEAS-2B cells grown for 6 months in 10% CSE or 0% CSE (control). Cells were stained with Mitotracker DeepRed and (3D) images were taken on a Leica AOBS confocal microscope.


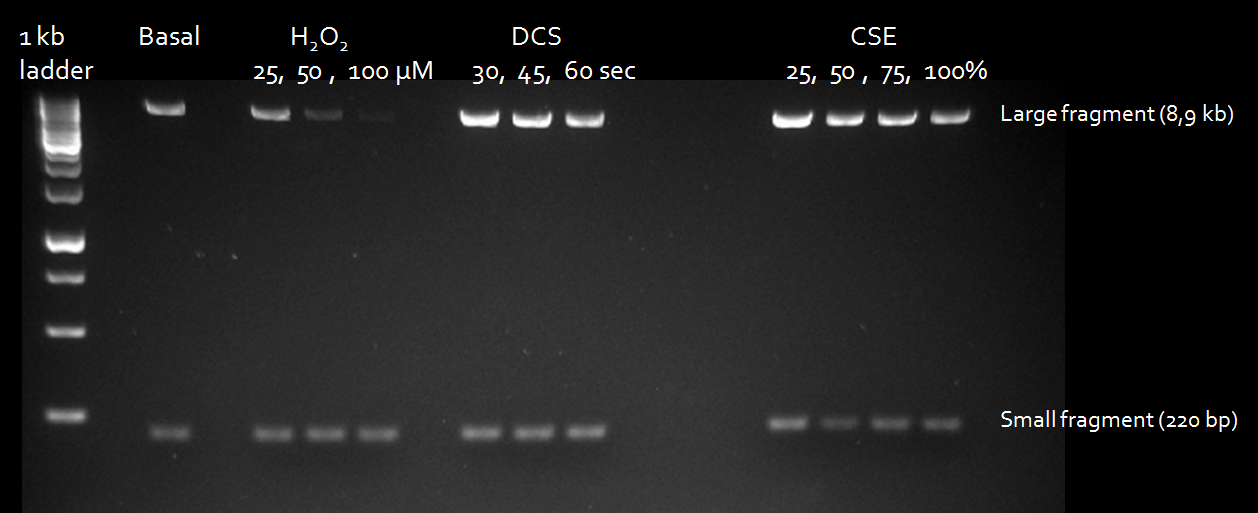


**S5. Oxidative stress induces mtDNA damage in BEAS-2B.** BEAS-2B were untreated (Basal) or treated with hydrogen peroxide (25, 50 and 100 µM), gas phase (Somborac-Bacura *et al*. *Exp Physiol*. 2013) cigarette smoke (CS: 30s , 45s and 60s) or cigarette smoke extract (CSE: 25%, 50%, 75% and 100%) for 5 hours. After total DNA isolation, two mitochondrial fragments were amplified using PCR and the short fragment (220bp) was used for normalization of mtDNA, and the large fragment (8,9 kb) was used for quantification of mtDNA damage upon analysis by gel electrophoresis (49). A dose-dependent damage of mtDNA after short-term treatment with hydrogen peroxide, gas-phase CS and CSE was observed.

**S6. PINK1 expression is not altered in long-term CSE-exposed BEAS-2B cells .** BEAS-2B cells grown for 6 months in 0%, 2.5% or 10% CSE. mRNA expression of PINK1 IL8 (n=4) mRNA expression was detected by qPCR and related to the housekeeping genes.. ΔCt values are shown (lower values reflect higher expression) and median interquartile ranges (IQR) are indicated.
